# Cervical Dystonia and Executive Function: A Pilot Magnetoencephalography Study

**DOI:** 10.3390/brainsci8090159

**Published:** 2018-08-22

**Authors:** Abhimanyu Mahajan, Andrew Zillgitt, Abdullah Alshammaa, Neepa Patel, Christos Sidiropoulos, Peter A. LeWitt, Susan Bowyer

**Affiliations:** 1 Department of Neurology, Henry Ford Hospital, 2799 W. Grand Boulevard, K-11 Detroit, MI 48202, USA; zillgit1@gmail.com (A.Z.); aalsham1@hfhs.org (A.A.); npatel20@hfhs.org (N.P.); plewitt1@hfhs.org (P.A.L.); sbowyer1@hfhs.org (S.B.); 2 Department of Neurology and Ophthalmology, Michigan State University, East Lansing, MI 48824, USA; ccsidirop@gmail.com

**Keywords:** cervical dystonia, functional imaging, magnetoencephalography, botulinum toxin, executive function

## Abstract

Background: Cervical dystonia (CD) patients have impaired working memory, processing speed and visual-motor integration ability. We used magnetoencephalography (MEG) to investigate changes in cerebral oscillations in CD patients during an executive function test, before and after administration of botulinum toxin. Methods: MEG data were collected from five CD patients while they performed a visual continuous performance task (CPT), before and after they received a botulinum toxin injection. MEG data was also collected on five controls matched for age and gender. Coherence source imaging was performed to quantify network connectivity of subjects. Results: Controls demonstrated two errors with visual CPT; CD patients demonstrated six and three errors pre- and post-botulinum toxin respectively. After botulinum toxin, mean time from cue to correct response was 0.337 s in controls, 0.390 s in patients before botulinum toxin injection, and 0.366 s after the injection. Differences in coherence between controls and patients were found in the following brain regions: Fronto-frontal, fronto-parietal, fronto-striatal, fronto-occipital, parieto-parietal and temporo-parietal. Intrahemispheric and interhemispheric networks were affected. Post injection, there was minimal change in coherence in the above-mentioned networks. Discussion: Neuropsychological testing suggests difference in coherence in frontal circuits between CD cases and controls during the visual CPT, which may reflect subjects’ increased difficulty with the task. Botulinum toxin is associated with minimal improvement with executive function in CD.

## 1. Introduction

Dystonia is defined as a variable pattern of sustained involuntary movement characterized by over-active muscle contractions, which are often associated with voluntary movement [1]. While its diagnosis is based on motor manifestations, dystonia originates as a brain network disorder with clinical manifestations extending beyond motor symptoms to effects on cognition [2,3].

Various aspects of altered cognition have been described in dystonia. For example, Romano et al. showed impairment in working memory, processing speed, visual motor ability and short-term memory in patients with cranio-cervical dystonia [4]. Testing for executive dysfunction did not yield abnormal findings in another study of dystonia patients [5].

In addition to neuropsychological testing, functional connectivity has been a topic of interest in dystonia. Abnormal fronto-striatal connectivity and cerebellar involvement has been reported in hereditary dystonia using 18F-fluoro-deoxyglucose positron emission tomography (FDG-PET) and diffusion tensor imaging (DTI) in the resting state [6]. Using resting state functional MRI (fMRI), one study showed altered functional connectivity within the sensorimotor, the executive control, and the primary visual networks in cervical dystonia (CD) patients [7]. Recently, fMRI studies by Burciu et al. showed differences between CD patients and controls in the primary somatosensory cortex, cerebellum, dorsal premotor and posterior parietal cortices, and occipital cortex. In the latter study, several correlations were found between clinical features of dystonia and functional activity in both the somatosensory cortex and the cerebellum [8].

Magnetoencephalography coherence source imaging (MEG-CSI) has also been used to investigate CD [9,10]. Our previous MEG-CSI study demonstrated difference in resting-state coherence between controls and CD patients in the fronto-striatal region, but not in occipito-striatal, parieto-striatal and striato-temporal networks [9].

Botulinum toxins function by cleaving members of the soluble *N*-ethylmaleimide-sensitive-factor attachment receptor (SNARE) protein family, which are responsible for mediation of the fusion of synaptic vesicles with the neuronal presynaptic plasma membrane [11]. Proteolysis of these proteins therefore interrupts neurotransmission and leads to flaccid paralysis of the muscle. Given botulinum toxin’s efficacy and safety in appropriate dosage, it has been deemed to have level A efficacy for CD [12].

While studies with fMRI and other functional imaging techniques provide insight into CD in a resting state, not much is known about task-specific changes in functional connectivity in CD patients. In this current project, our aim was to use MEG CSI to investigate changes in CD patients’ cerebral oscillations at the network level during performance of a task assessing executive function.

## 2. Methods

MEG data were collected from five patients with CD (Table 1), before and after standard therapeutic botulinum toxin injections (which had also been used previously).

To be included in the study, patients needed to have gained significant benefit from the previous injections. MEG studies were conducted between 2 and 3 weeks post injection. For comparison, MEG data was also collected from five age- and gender-matched controls lacking any neurological disorder. The controls were not injected as part of this study.

MEG recordings were conducted while patients and controls were performing a visual continuous performance test (CPT). In this task, random letters were shown briefly for 150 ms with a 1.8 s inter-stimulus interval (100 trials). Participants were instructed to respond as quickly as possible by pressing a button when the “X” target stimulus followed the “A” cue stimulus [13]. This CPT task serves as a measure of executive function, and especially selective attention [14].

Oscillations of spontaneous neural activity can be detected by MEG and seen in the in MEG waveform recordings. These neuronal oscillations can be quantified by applying a short-time Fast Fourier Transform (stFFT). After transformation to a time-frequency representation, the strength of network interactions can be determined by calculating the coherence, which is a measure of synchrony between signals from different regions for each FFT frequency. Using MEG-Coherence Source Imaging (MEG-CSI), Elisevich et al. found regions of high coherence primarily located in the cerebral cortex and localized in the epileptogenic regions, and a seizure-free outcome was associated with removal of these highly coherent networks [15,16,17].

A detailed description of the MEG based methodology used in this study has been previously published by our lab in a resting state MEG study of CD patients [9].

This study was performed in accordance with the ethical standards laid down in the 1964 Declaration of Helsinki and its later amendments. Permission was received from Henry Ford Hospital Institutional Review Board (IRB # 9460) in April 2015 and last renewed in April 2018. All persons gave their written informed consent prior to their inclusion in the study. No patient-identifiable information was included in the text.

## 3. Results

**Figure 1:**
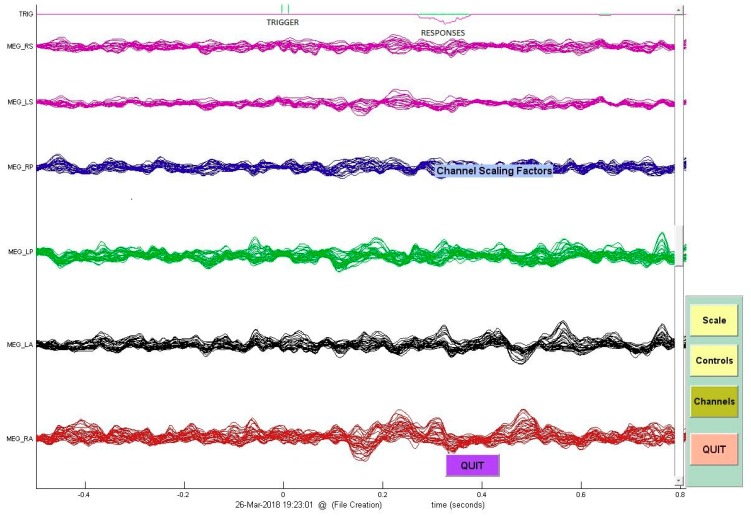
As part of the visual continuous performance test (CPT) test, subjects are instructed to respond as quickly as possible by pressing a button when the “X” target stimulus followed the “A” cue stimulus. This figure represents the trigger/stimulus and the latency in response (pressing the button) by the subject.

During the visual CPT, control subjects made two errors in total. CD patients made six errors during the pre-botulinum toxin treatment recording and three errors post-botulinum toxin. The average time from visual cue to correct response was 0.337 seconds in controls, 0.390 seconds in CD patients pre-botulinum toxin and 0.366 seconds in CD patients post-botulinum toxin (Figure 1).

**Figure 2:**
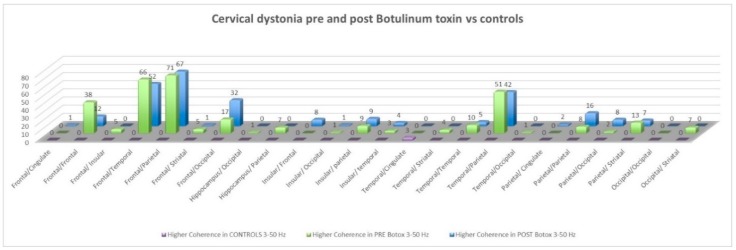
Difference in coherence between patients and controls pre- and post-botulinum toxin. The number signifies the number of significant networks with a large effect size within a given pathway.

Prior to botulinum treatment, out of a total of 1431 networks, 162 interhemispheric networks and 126 intrahemisheric networks (75 right and 51 left) were statistically significantly different between the two groups (*p* < 0.05). Pathways with a statistically significant difference in coherence between patients and controls were the following: Fronto-frontal, fronto-parietal, fronto-temporal, fronto-striatal, fronto-occipital, parieto-parietal, parieto-striatal and temporo-parietal (Figure 2).

**Figure 3:**
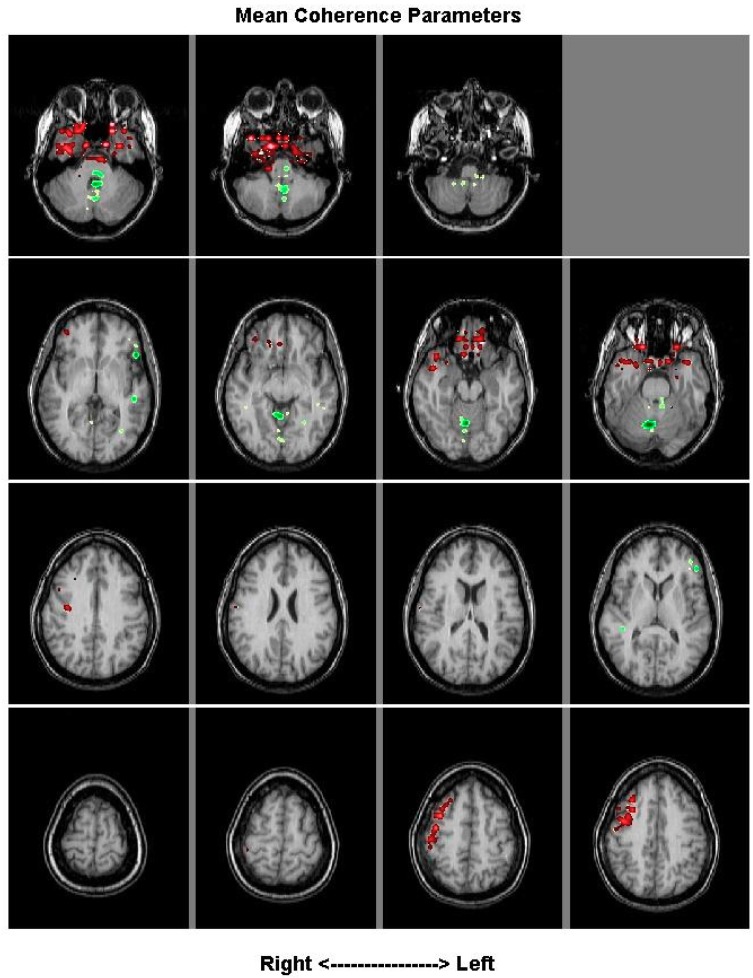
Patient 4, Magnetoencephalography (MEG) images with visual CPT, pre and post botulinum toxin. Regions of the brain that are statistically significantly different in this subject pre- (green) and post- (red) botulinum toxin medication. Red areas indicate where more coherent activity was seen after treatment. Green areas had higher coherence prior to treatment.

The averaged coherence across 1 patient pre-treatment minus the average of their coherence post-treatment indicated significant changes to coherence took place in their inferior frontal lobe (red) (Figure 3).

After treatment, out of a total of 1431 networks, 193 interhemispheric networks and 105 intrahemisheric networks (24 right and 81 left) were statistically significantly different between the two groups. Coherence between the same pathways remained statistically significant (Figure 2).

On comparing patients post- and pre-botulinum toxin, out of a total of 1431 networks only five networks were statistically significantly different: fronto-parietal, fronto-cingulate, fronto-occipital, parieto-insular and parieto-temporal.

Coherence was lower in the alpha band pre-treatment compared to post-treatment in three of the five CD patients. Beta and Gamma band coherence was lower in three of the five patients, post-treatment (*p* < 0.05).

## 4. Discussion

This is the first study using MEG-CSI to assess task specific network connectivity in patients with CD and to assess the impact of botulinum toxin treatment giving symptomatic benefit for dystonic features. Our study reiterates the importance of frontal networks in the cerebral cortex in tasks that assess attention and other components of executive function. It further indicates the importance of the parietal lobe in selective attention.

As previously stated, the spectrum of executive function in dystonia has been of great interest, even though studies to date have reported inconsistencies [4,5]. Testing for executive function in a disorder affecting motor function poses a research challenge in that most tests for executive function use time-to-completion as a metric, which might be affected by the confounding feature of motor impairment in dystonia. Another challenge is the difficulty in differentiating executive dysfunction attributable to physiological aging from neurodegeneration in older patients experiencing movement disorders like CD [18]. One strength of our study lies in assessing changes in task-related connectivity [19]. Our study shows a difference in the networks thought to be involved with executive function in CD patients, as compared to age- and sex-matched controls.

Our previous study assessing changes in coherence using resting state had shown that compared to controls, CD patients showed a state of lower amplitude and frequency of synchronicity, and a lower frequency of synchronicity of oscillating neurons, or coherence [9]. With task specific testing, controls showed increased coherence in one pathway involving the cingulate gyrus, associated with decision-making. CD patients showed diffusely increased coherence compared to controls, especially in the frontal networks. We hypothesize that the diffuse increase in coherence in CD patients compared to controls perhaps indicates reduced efficiency/ difficulty in performing the go-no-go task of visual CPT [20]. This is further suggested by increased reaction time and greater number of errors in patients as compared to controls. The dissimilarity in resting state and task specific differences between CD patients and controls offers an interesting insight into the pathophysiology of CD.

Our study is also the first to assess the effect of botulinum toxin treatment on network connectivity while performing a task assessing executive function using MEG-CSI. Previously, we showed that efficacious botulinum toxin use is associated with a change in coherence in specific pathways in the resting state, suggesting a role of sensorimotor integration in clinical benefit [9]. Our current study shows a statistically significant change in coherence, albeit small, during the visual CPT in CD patients with botulinum injection. Increased coherence is further suggested by patients’ improvement in average reaction time and reduced number of errors. However, given the small magnitude of observed changes, we advise caution in interpretation of these findings. Moreover, our study was not designed to assess the effect of task-specific change in coherence in executive function-related frontal networks on improvement in motor function or neuropsychological testing, which remains a question for future studies.

Pallidal oscillatory activity in theta-alpha range correlates with involuntary muscle activity in dystonia [21]. In their study on patients with CD, Neumann et al. showed that theta peak power in the pallidum correlates with severity of the clinical symptoms in dystonic patients, with the maximum theta peak amplitude correlating with clinical improvement with DBS [22].

In our study, our sample size precluded us from commenting on spectral signals in controls compared to CD patients and with botulinum toxin injection.

A significant contribution of our study is its use of MEG-CSI as a means of imaging brain function. This technique offers high temporal and spatial resolution and provides a direct measure of neuronal activity, unlike PET and fMRI, which measure changes in metabolic responses and blood flow and offer less temporal resolution [23]. The magnetometer-type system that we use in our MEG lab has better resolution for deeper structures than other models of the MEG scanner.

Our study has several limitations. We did not employ rating scales to assess changes in motor improvement in CD at the time of MEG-CSI testing. The control subjects did not receive saline injections as a part of the protocol to act as a control for the post-injection CD patients. While an independent component analysis was carried out to filter out heart sounds and other noise, it is possible that some movement artifacts might have partly confounded the results. While we looked at 1431 network connections per patient, our sample size of five subjects in this pilot study limits the generalizability of our results. A larger sample size is needed to carry out a more thorough comparison of neuropsychological testing and MEG-CSI findings. A larger number of participants would further allow us to characterize potential difference in coherence spectra between controls and patients, and with and without botulinum toxin.

## 5. Conclusions

Our pilot study, based on MEG data on five CD patients and age and sex matched controls predominantly shows a difference in coherence in the frontal circuits, those associated with executive functioning, during the visual CPT. This may reflect increased difficulty with the task in CD patients, which is also suggested by neuropsychological testing. Using a larger sample size, the difference in MEG coherence and spectral signals, between CD patients and controls, at resting state and with specific cognitive-tasks needs to be explored further.

## Figures and Tables

**Table 1:** Patient demographics and clinical characteristics.

	Patient 1	Patient 2	Patient 3	Patient 4	Patient 5
Age (years)	53	53	55	60	32
Gender	Female	Female	Male	Female	Female
Onset of symptoms	January 2016	February 2017	July 2015	March 1996	November 2015
Diagnosis	Cervical dystonia	Cervical dystonia	Cervical dystonia	Cervical dystonia	Cervical dystonia
Pharmacotherapy	Botulinum toxin type A	Botulinum toxin type A	Botulinum toxin type A	Botulinum toxin type A	Botulinum toxin type A
Description of dystonia	“right sided rotation and left sided bending”	“left torticollis and slight anterocollis with a slight no-no dystonic head tremor”	“Left torticollis with mild retrocollis and left shoulder elevation with dystonic tremor”	“right laterocollis and left torticollis with dystonic tremor”	“right torticollis with mixed “yes-yes” and “no-no” head tremor”
Type A toxin administered	150 units	250 units	280 units	200 units	280 units
